# Experimental Study on the Shear Behavior of GFRP–Concrete Composite Beam Connections

**DOI:** 10.3390/ma13051067

**Published:** 2020-02-28

**Authors:** Jin Di, Lu Cao, Jiahao Han

**Affiliations:** 1 School of Civil Engineering, Chongqing University, Chongqing 400045, China; lucao1996@126.com (L.C.); hanjiahao@longfor.com (J.H.); 2 Key Laboratory of New Technology for Construction of Cities in Mountain Area, Chongqing University, Chongqing 400045, China

**Keywords:** GFRP–concrete composite beam, push-out tests, shear connection, perforated plate, shear stiffness

## Abstract

Monotonic push-out tests were carried out on 11 specimens having high-strength bolt, T-type perforated plate, or slot-type perforated plate connections to investigate the influence of different connection types on the interface performance of glass fiber reinforced polymer (GFRP)–concrete composite beams. The effects of the number of rows and spacing of high-strength bolts on the failure mode, load–slip relationship, and shear capacity were analyzed. The effects of the number and spacing of holes in the perforated plates, and the inclusion of transverse rebar were analyzed. The results show that the failure mode of the bolt specimens is brittle failure and the perforated plate is ductile failure. The single bolt connection has the lowest ultimate bearing capacity, while the single T-shaped and slotted perforated plates are 170% and 270% times greater. The rows and spacing have no difference in bolts. The perforated plate gradually decreased with an increase in rows and gradually increased with an increase in spacing. The transverse rebar can increase the ultimate bearing capacity and ductility in the plastic stage for perforated plate. Accordingly, there are good choices for interface connectors for GFRP–concrete composite beams, while bolt connectors need to be carefully chosen.

## 1. Introduction

Fiber reinforced composite (FRP) is a new type of material with high performance, which is composed of fiber material and matrix material. Glass fiber reinforced polymer (GFRP) refers to the resin matrix that is reinforced by glass fiber. Fiber-reinforced polymer (FRP)–concrete composite beams are a new type of structure that can combine the respective advantages of the two materials, including concrete’s compressive strength and FRP’s high tensile strength [1]. There are many advantages with the FRP-concrete composite beams, referring to the increase of the flexural stiffness, the increase of the structures strength capacity and reducing the structures deformation, better corrosion resistance, and so on [2,3,4]. The connection between the concrete slab and FRP profile is the key factor affecting the bearing capacity of FRP–concrete composite beams [5]. In the steel-concrete composite structure, the shear connectors include reinforcement connectors, high-strength bolt connectors, section steel connectors, stud connectors, perforated steel plate connectors, and other connection types [6,7]. Refer to steel-concrete composite structure connector and consider the characteristics of FRP, researchers have proposed several connection methods, such as natural friction, adhesive joints, expansion agents, bolt connections, and perforated FRP connections. Of these, the natural friction connection only depends upon the interfacial adhesion and friction force that formed with the FRP profiles when pouring concrete to provide the bearing capacity, which is poor [8]. Adhesive joints are easy to construct, but the bearing capacity of the adhesive layer is small and unstable, which makes it difficult to meet the requirements for the large interface shear force of the composite structure. It often works together with a mechanical joint [9]. The use of an expansion agent can closely combine the FRP and concrete as a result of the pressure formed after the cement base material solidifies; this method has a high connection efficiency, but the process is complex and the quality is difficult to guarantee. The bolted specimens can provide a considerable bearing capacity and they are widely used; however, the slip stiffness of this method is insufficient. In the initial stage of loading, it is necessary to overcome the opening clearance to increase the component slip in the early stage [10]. Perforated FRP connectors have great development potential with high shear rigidity and shear capacity, good fatigue resistance, simple construction, and reliable quality [11].

Over the last two decades, many studies have investigated the connections between FRP and concrete. Canning et al. [12] carried out an experimental study on six different types of FRP–concrete composite structures. The results showed that the bending rigidity is the highest for specimens that are connected while using dented FRP plates, while that of specimens that are connected with bolts is lowest. Nordin et al. [10] used beam test to compare the shear resistance of the two methods of bolt and epoxy resin adhesive. The test results show that the two shear connection methods can effectively transfer the shear of the composite interface, but the bending rigidity of the beam bonded with epoxy resin is greater. Wang et al. [13] investigated the FRP–concrete interface, and the results showed that the length of the bonded coarse sand, the type of epoxy adhesive, and the strengthening of the FRP cloth affected the interface bond strength. Feng et al. [14] studied the shear resistance on four kinds of shear connectors, which included adhesive, stud, FRP I-shaped, and wave shaped formwork. It is found that the studs have the highest bearing capacity and the adhesive have the best anti sliding rigidity. Wang et al. [15] carried out beam tests of FRP bolt connections and evaluated the influence of the FRP material characteristics, laying mode, and interface connection to elucidate the failure mode of the FRP profile–concrete composite beam; a simplified calculation method for the ultimate bearing capacity of the composite beam was also presented. Xue et al. [5] carried out experimental research on the shear key and adhesive of the FRP perforated plate. The results show that: the failure mode of shear test piece with FRP perforated plate connection is shear failure at the root of FRP perforated plate; the failure mode of shear test piece with epoxy resin adhesive is divided into coagulation main cohesive failure and adhesive layer failure. Zou et al. [16,17,18] analyzed the difference in the bearing capacity and slip stiffness between the specimens that were connected with perforated plates and bolts. The results showed that the bolted specimen had a higher bearing capacity, but its slip stiffness was far lower than that of the perforated plate specimen. Subsequently, 14 bolt specimens were tested, and the differences between ordinary bolts, high-strength bolts, ordinary concrete, and high-performance concrete were compared and analyzed. The load–slip curve for the bolt specimens was proposed as a three-fold model.

In general, experimental studies on shear connectors in FRP–concrete composite structures are still lacking and they have largely focused on the cementation and bolt connection, while research on perforated plate specimens is still in its infancy. Accordingly, limits the use of FRP–concrete composite structures. In this study, an experimental investigation of the shear behavior between GFRP and concrete was carried out. The interconnection slip behavior of the steel bolts and perforated plates is compared based on the load–slip curves, shear capacity, and shear stiffness.

## 2. Specimen Fabrication and Test Setup

### 2.1. Test Specimens and Materials

**Figure 1:**
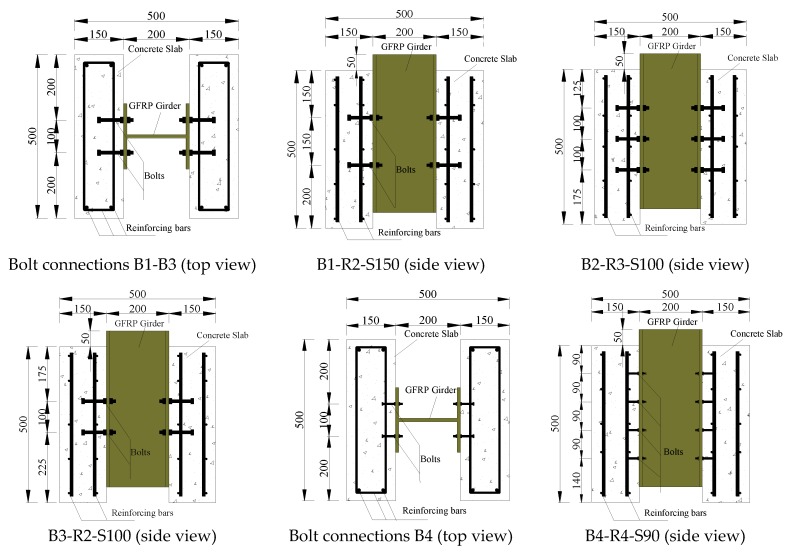

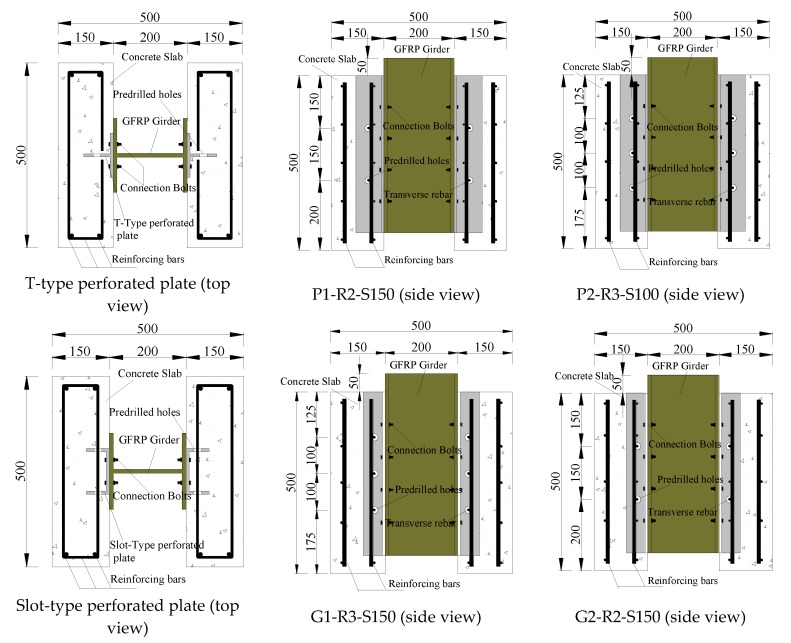
Composition of the push-out specimens (units: mm).

The push-out test method is used in this study because there is no standardized method to test FRP shear connectors. A total of 11 specimens were designed in accordance with Eurocode 4 [19], including high-strength bolt connections, T-type perforated plates, and slot-type perforated plates. The push-out test specimens included a short segment of FRP I-girder that was connected to two small-scale concrete slabs by shear connectors. The dimensions of GFRP I-girder is 200 mm × 200 mm × 10 mm. The dimensions of T-type perforated plates is 120 mm × 60 mm × 8 mm and slot-type perforated plates is 120 mm × 60 mm × 8 mm. The T-type perforated plates and slot-type perforated plates are FRP pultruded profiles. Table 1 and Figure 1 provide the parameters of the test specimens.

The mechanical properties of the concrete were determined in accordance with the relevant standard for the test method for the mechanical properties of ordinary concrete (GB/T 50081-2002) [20]. The cube strength was 52.6 MPa and the Young’s Modulus was 34.9 GPa. The reinforcing bars are made of ribbed steel, with a yield strength of 400 MPa. The material properties of GFRP provided by the manufacturer. It corresponds to the Pultruded fiber reinforced polymer composites structural profiles (GB/T 31539-2015) M25, as specified [21].

### 2.2. Specimen Fabrication

**Figure 2:**
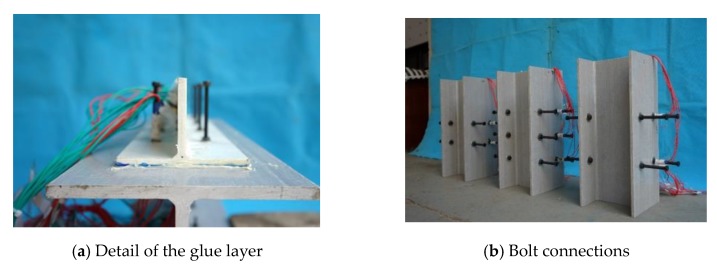

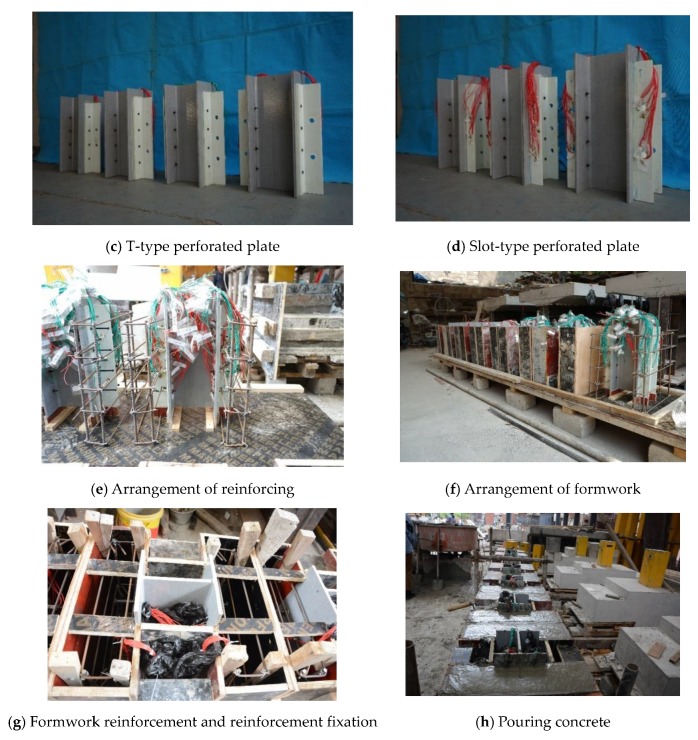
Images of fabricated specimens.

First, the GFRP surface was sanded and the contact surface was rinsed with acetone. Subsequently, the matched glue solution was applied on both sides of the surface of the test piece. After applying the glue, but before the glue had solidified, a torque wrench was used to tighten the connecting bolts. Figure 2 shows images of the fabricated specimens. Foam was placed under the test piece to ensure that the load was borne by the connecting piece. When pouring the concrete, a wooden formwork was used to reinforce the concrete slabs on both sides to avoid deformation of the formwork during pouring and vibrating.

### 2.3. Test Setup, Loading Procedure, and Instrumentation

**Figure 3:**
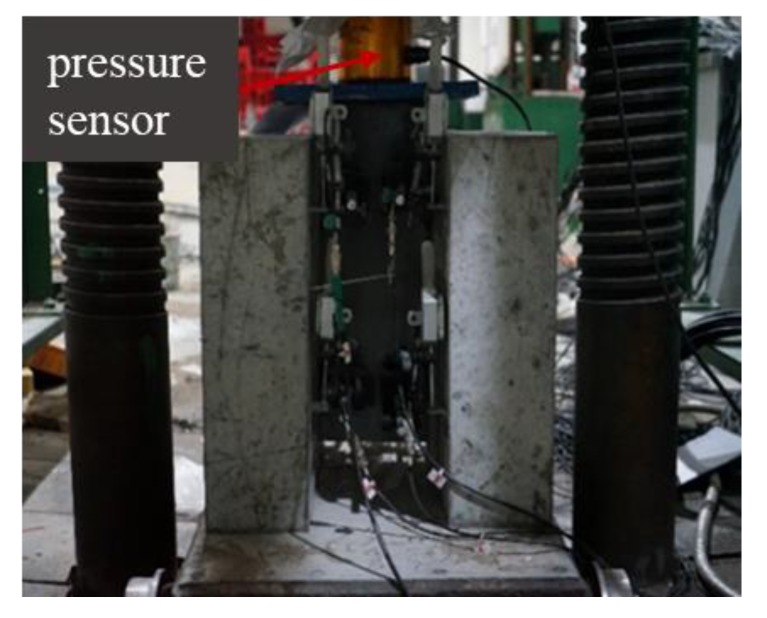
Loading device used for the push-out tests.

**Figure 4:**
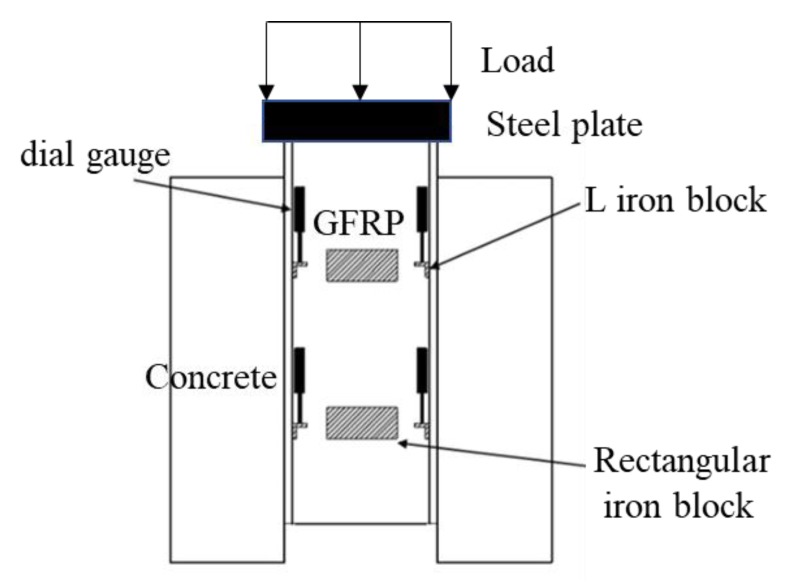
Setup of the push-out tests.

All of the push-out tests were performed while using a force-controlled 500 t loading apparatus (Figure 3). A steel plate was placed on the upper part of the main beam to ensure that the load was evenly applied on the I-shaped section, such that the connection was in a state of pure shear stress. A layer of fine sand was arranged at the bottom of the specimen to ensure a uniform force on the left and right concrete slabs in order to avoid eccentric compression of the specimens. It was also necessary to ensure that the GFRP beam and the perforated plate were in a state of complete void before component installation. The force control was used in the initial loading stage, which had a loading rate of 1 kN/s and 10 kN/grade. When the load displacement exceeded 0.5 mm, the displacement was subsequently controlled at 0.5 mm/grade until the complete failure of the specimen. The main results obtained from these tests are the load–slip curves of the components, the cracking state of the concrete, and the ultimate bearing capacity of the specimens. For this purpose, eight dial gauges were used to measure the displacement of the connecting parts; a rectangular steel plate and L-steel block were pasted on the web of the GFRP and the concrete, respectively, to fix the dial indicator init. In addition, a magnetic suction seat fixed displacement meter was used to measure the relative slip (Figure 4), while the pressure was measured while using a pressure sensor.

**Figure 5:**
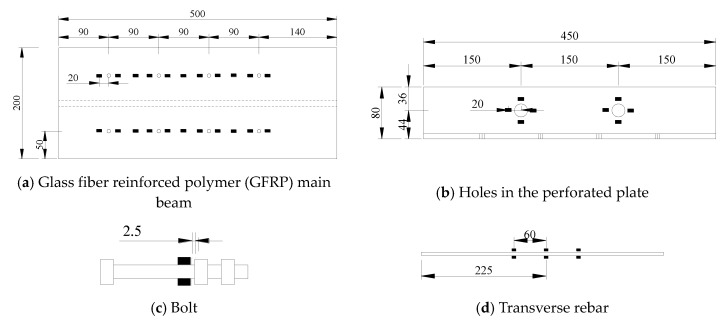
Strain measurement points. (units: mm).

The strains near the main GFRP beam, holes in the GFRP, bolts, and transverse rebar were measured while using electrical resistance gauges during the loading process, as shown in Figure 5.

## 3. Push-Out Test Results and Discussion

### 3.1. Failure Modes

**Figure 6:**
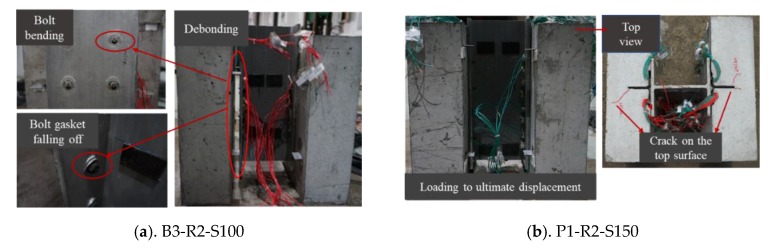

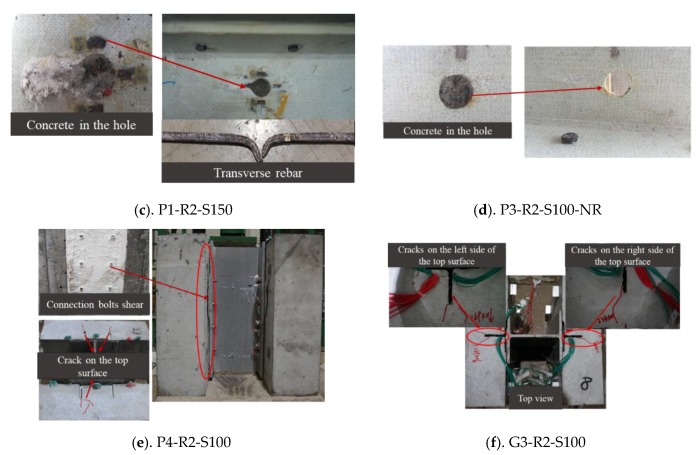
Failure of specimens.

The failure modes of the four high-strength bolts are all shear failure after the concrete is crushed based on the test results. Specimen B3-R2-S100 is taken as an example to illustrate this failure mode When the load reaches 40 kN, the load decreases slightly. Before reaching the ultimate load, the load increases linearly, and the interface between the GFRP main beam and concrete peels off. When the load reaches 250 kN, the bolt is obviously bent, and the upper end of the gasket is separated. The displacement of the main beam gradually increases, and the upper loading surface exhibits significant compression deformation. Two bolts on one side suddenly fractured at approximately 292 kN. Figure 6a shows this failure mode.

The failure modes of specimens P1-R2-S150 and P2-R3-S100 are shear failures of the concrete tenon and the transverse rebar. Taking P1-R2-S150 as an example to illustrate this failure mode, when the load reaches 240 kN, the GFRP tearing sounds can obviously gradually begin to be heard and, as the load increases, the fracture sound becomes more intense. When the load reaches 245 kN and 265 kN, the first and second cracks appear on the right and left sides of the upper concrete, respectively. Observing the failure mode of the internal structure of the P1-R2-S150 test piece, it can be seen that the concrete tenon and the transverse rebar are severed. The cutting failure of the perfobond GFRP occurs along the direction of the opening line. The GFRP in the failure area is pressed into a white powder form, and the GFRP cracked along the direction of the fiber lines between two pre-drilled holes. Figure 6b shows the failure mode and Figure 6c shows the concrete in the hole. For the P3-R2-S100-NR specimen with no transverse rebar, the failure process is similar to that for P1-R2-S150. The concrete tenon is sheared, and there is no shear failure around the GFRP hole. Figure 6d shows the damage at the perfobond GFRP.

The failure mode of specimen P4-R2-S100 with a T-shaped perforated plate is the separation of the interface between the GFRP beam and concrete slab. P4-R2-S100 is taken as an example for illustrating this failure mode. In the beginning, the sound of the GFRP tearing could be clearly heard until the load reached 90 kN. When the load reached 169 kN, the first crack appeared on the left side of the concrete slab and then expanded. When the load reached 234 kN, a second crack appeared on the right side of the concrete slab. The load stopped increasing once it reached 253 kN; when the load decreased to 151 kN, the sound of the reinforcement fracturing could be heard, and the GFRP and concrete were obviously separated. Figure 6e shows this failure mode.

There are two failure modes for the slotted perforated plate, G1-R3-S100 and G2-R2-S150; however, these are similar to the failure mode for the first T-shaped perforated plate and, thus, will not be discussed further here. Another failure mode is the failure of the bond layer between the perforated plate and GFRP beam, which is illustrated by specimen G3-R2-S100. During the test, the sound of a GFRP connector tearing could be clearly heard as a result of shear failure. Finally, the whole concrete part on one side fell off, the bond layer between the opening plate and the GFRP beam was damaged, and the fiber of the GFRP was pulled off. It was found that there was no coarse aggregate in the concrete tenon, and it was sheared together with the transverse rebar during the loading process. Figure 6f shows this failure mode.

### 3.2. Load–Slip Relationships

The displacement value in the figure is the average value that is recorded by four dial indicators at the lower end of the test component to reduce the influence of the component manufacturing error.

#### 3.2.1. Bolt Connectors

**Figure 7:**
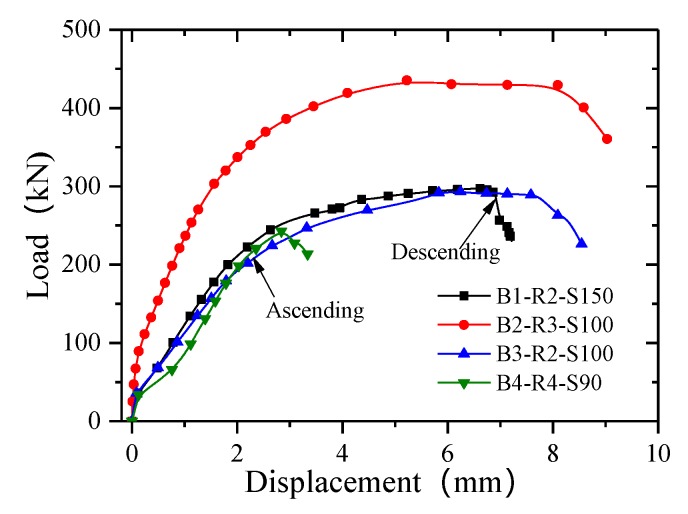
Load–slip curves for the bolt connections.

Figure 7 presents the load–slip curves for the bolt connections. The load–slip curves can be divided into two phases: ascending and descending. There is almost no slip in the early stages, owing to the contribution of the bonding. After debonding occurs between the GFRP girder and the concrete slabs, the slip curve exhibits linear growth with increasing load. When the elastic limit load is reached, the curve gradually exhibits nonlinear growth, and the rate at which the load increases gradually slows, while the amount of slip increases continuously. The specimen is damaged when the bolts on one side suddenly fracture. The load–slip curve then drops sharply and enters a decreasing stage.

#### 3.2.2. T-Type Perforated Plates

**Figure 8:**
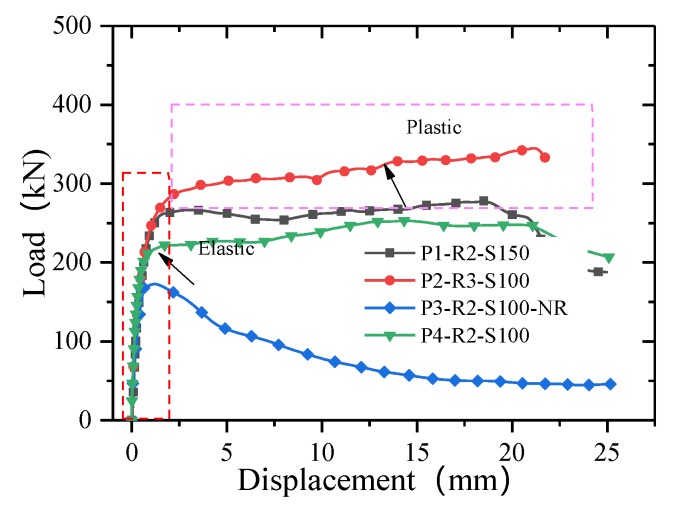
Load–slip curves for the T-type perforated plate connections.

Figure 8 presents the load–slip curves for the T-type perforated plates. Initially, owing to the initial bond force between the GFRP and concrete, the stiffness of the connection is high. With increasing load, the initial bond force disappears, and the interface shear force is completely borne by the concrete and the transverse rebar in the dowel holes; as a result, the specimen slip has a linear relationship with the load. Finally, when the plastic stage is reached, the load changes little after a slight strengthening section appears in the curve, but the displacement continuously increases. The specimen failed immediately when the transverse rebar was severed.

**Figure 9:**
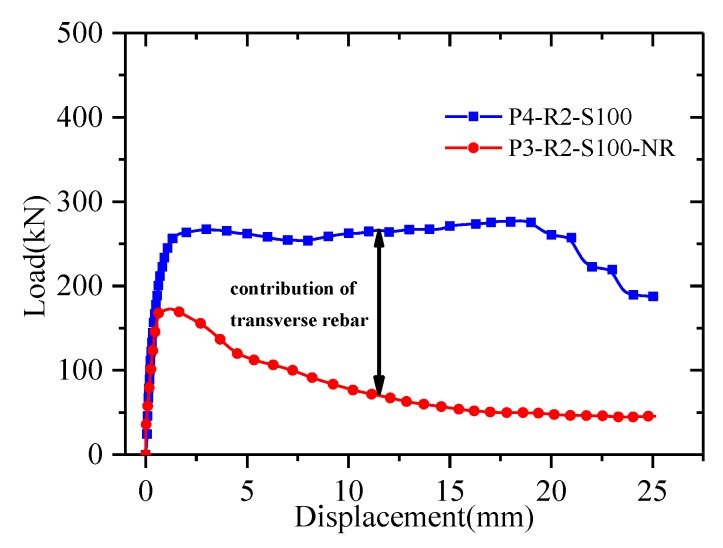
Contribution of the transverse rebar.

Figure 9 shows a comparison of the effectiveness of the perforated reinforcement. The T-type perforated plate connection without a transverse rebar is similar to the above specimens with transverse rebar in the elastic stage, and there is no yield platform before the load reaches the ultimate state. After reaching the ultimate load, slip occurs rapidly, and the load continuously decreases and does not stabilize until the specimen loses its bearing capacity. When comparing the load–displacement curves for specimens P3-R2-S100-NR and P4-R2-S100 can reveal the contribution of the transverse rebar to the bearing capacity. The transverse rebar provides 43.18% of the ultimate bearing capacity and ductility in the plastic stage.

#### 3.2.3. Slot-Type Perforated Plate

**Figure 10:**
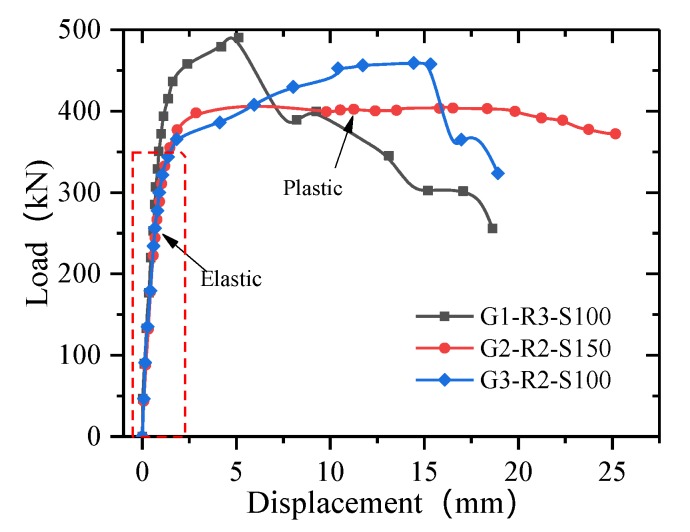
Load–slip curves for the slot–type perforated plate connections.

Figure 10 presents the load–slip curves for the slot-type perforated plates. It can be seen that the load–slip curve can be divided into two phases. In the first phase, the specimen displacement has a linear relationship with the load. With increasing load, the displacement changes little, and the shear connectors exhibit high shear stiffness. The second stage is a nonlinear elastic–plastic stage, in which the displacement increases nonlinearly with an increasing load. In this stage, the load increases slowly, the displacement increases significantly, and the shear rigidity of the specimen decreases continuously.

### 3.3. Load Versus Longitudinal Strain Curves

**Figure 11:**
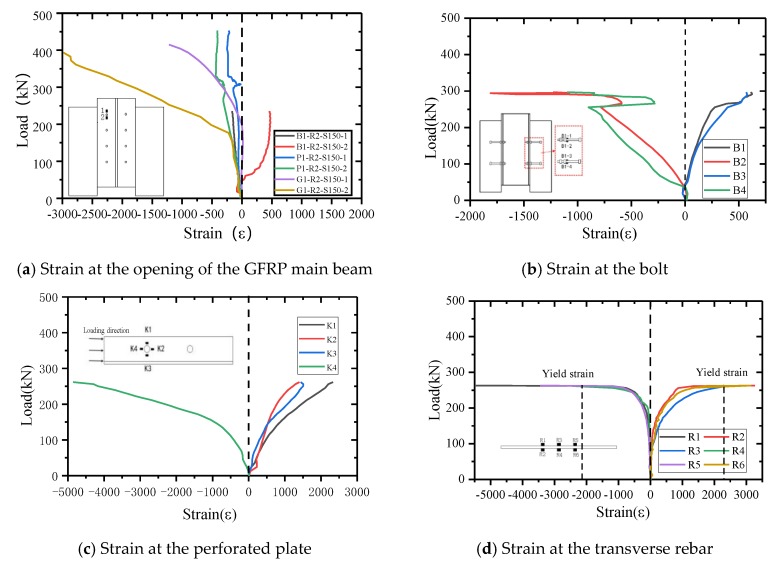
Load versus longitudinal strain curves.

When comparing the strains of the main beam with the bolted connection and the GFRP perforated plate connections can reveal the difference in the stress distribution between the bolted connection and the hybrid connection (bonded and bolted). The perforated plate and the GFRP main beam enhance the overall performance of the connection through the hybrid connection. Figure 11a shows the stress distribution for the three types of connectors at the opening of the main beam. For comparison, the representative strains at the openings of the main beam in specimens B4-R4-S90, P1-R2-S150, and G1-R2-S150 are shown. B4-R4-S90 adopts the same bolts as the connecting bolts in the perforated plate connections. The strains exhibit a linear increase with increasing load at the beginning of loading. The opening of the main beam of the B1-R2-S150 specimen is under compression at the upper edge and under tension at the lower edge. The stress of the main GFRP beams in the P1-R2-S150 and B1-R2-S1500 specimens is uniform, and the upper and lower edges of the opening are all under compressive strain. Thus, it is recommended to use hybrid connections (bonded and bolted) to improve the overall performance of the connection piece.

For an interface connector, it is helpful to know the distribution of the interface shear force and the redistribution of stress in the plastic state by elucidating the variation in the stress with the load through strain. When the load is less than 50 kN, the strain measurement point exhibits little change, as shown in Figure 11b (specimen B1-R2-S150). At this stage, the bolt specimen is in the contact stage, and the load is mainly borne by the interface bond force. When the load is greater than 50 kN, the total strain basically exhibits linear growth. When the load is close to the ultimate load, the strain no longer varies linearly. Based on the stress characteristics of the test piece, it can be determined that the upper edge of the bolt is under tension and the lower edge is under compression. The test results indicate that, in the process of loading, the upper edge of the bolt is under tension and the lower edge is under compression, thus verifying the strain results for each bolt.

It is necessary to ensure that the perforated plate does not undergo brittle fracture during loading. The perforated plate is on the shear plane, and the strain gauge can easily fall off or be damaged during the loading process. Here, the strain–load curve for specimen P1-R2-S150 in the elastic stage is considered. It can be seen from Figure 11c that measuring points K1–K3 are under tensile strain, while K4 experiences compressive strain near the loading end. Even in the elastic stage, the strain load curve around the opening still exhibits strong nonlinearity, which is a result of the anisotropy of FRP materials.

The transverse rebar model number is HRB400, and it has a diameter of 6 mm. When the strain reaches 2105 εu, the rebar is considered to enter the yield stage. Specimen P1-R2-S150 is considered as an example to illustrate the strain test results for the transverse rebar (as shown in Figure 11d). The results show that, from the beginning of loading to the end of failure, strain gauges R1, R4, and R5 are under tensile strain, while strain gauges R2, R3, and R6 are under compressive strain. These test results are consistent with the failure mode of the reinforcement. When the load reaches approximately 250 kN, the lower surface of the steel bar begins to yield, the tensile stress is significantly higher than the compressive stress, and the lower surface is in the tension bending state. When the load reaches approximately 260 kN, the lower surface of the perforated steel bar begins to yield, the tensile stress is greater than the compressive stress, and the upper surface is also in the tension bending state.

## 4. Calculation of the Bearing Capacity and Rigidity of the Interface Connections

### 4.1. Ultimate Shear Capacity of a Single Connection

It is assumed that every connector in the same specimen was subject to the same force. Thus, the shear capacity per bolt or concrete wedge can be given, as follows:

(1) $$F_{u}=\frac{P_{u}}{n}$$

where *P_u_* is the experimentally obtained maximum load of the specimens in the push-out tests and *n* is the number of connectors in the specimen. It stands for the number of high strength bolts and number of openings in the perforated plate respectively.

From Table 2, it can be seen that the bearing capacities of a single connection of specimens B1-R2-S150, B2-R3-S150, and B3-R2-S100 are almost the same. Thus, the bolt spacings of 150 mm and 100 mm have little impact on the bearing capacity of the connection pieces, and the influence of using either three rows or two rows on the bearing capacity of the connection pieces is also within 3%. The bolts used in B4-R4-S90 are connecting bolts in the perforated plate specimen, with a bolt grade of 12.9. Comparing the B1-R2-S150, B3-R2-S100, and B4-R4-S90 members reveals that, the higher the bolt grade is, the worse the bearing capacity of a single connection will be. When the bolt strength is low (i.e., the bolt is weak relative to the concrete slab), shear failure of the bolt specimens mainly occurs, and the shear capacity increases with increasing bolt tensile strength. However, when high-strength bolts are used, the concrete is destroyed first, and the bearing capacity is then reduced.

In contrast to the bolt specimens, the T-shaped perforated plate is more sensitive to the spacing and number of rows. When the spacing increases from 100 mm to 150 mm, the bearing capacity of a single connection increases by 11.11%. As the spacing increases, the stress diffusion at the opening on the compression side is more uniform and the bearing capacity increases. The bearing capacity decreases by 7.1% when the number of rows increases from two to three. This phenomenon is mainly attributed to the overlapping action zones in the specimens. When comparing the bearing capacities of single connections of specimens P1-R2-S100 and P3-R2-S100-NR reveals that the contribution of the perforated reinforcement to the bearing capacity reaches 43.18%. The transverse rebar plays an important role in the bearing capacity. Generally speaking, the spacing and number of rows affect the bearing capacity of T-shaped perforated plates. The spacing can be increased appropriately and the number of rows can be reduced in order to improve the working efficiency of these connectors.

As with the T-type connector, the spacing and number of rows have a significant influence on the bearing capacity of the slot-type perforated plates. When the number of rows increases from two to three, the bearing capacity of a single connection of G1-R3-S100 is 23% lower than that of G3-R2-S100. After increasing the number of rows, the stress superposition between the holes is not conducive to stress diffusion. Comparing G2-R2-S150 and G3-R2-S100 shows that, when the spacing increases from 100 mm to 150 mm, the bearing capacity increases by 13.8%, and the stress diffusion on the compression side of the opening is more uniform. As compared with the T-type perforated plate, the shear area of the slot-type perforated steel bar is greater, and the concrete tenon at the two holes provides shear and, thus, the ultimate bearing capacity of the slot-type perforated plate is approximately 60% higher than that of the T-type perforated plate.

### 4.2. Shear Stiffness

For the composite shear connectors, the shear stiffness is an important index for evaluating the performance of the composite structure, and can be represented by the secant slope of the load–slip curve. A comparison of the shear stiffness will be meaningless unless a uniform equation is used owing to the different spacings of the connectors along the beam direction. The secant stiffness corresponding to 70% of the ultimate bearing capacity is taken as the shear stiffness for the calculation of stud connections in Eurocode 4, as follows:

(2) $$K_{0.7P_{u}}=\frac{0.7P_{u}}{S_{y}}$$

where $K_{0.7P_{u}}$ is the slip modulus for a single concrete wedge or bolt, $S_{y}$ is the slip at a load of 0.7*P_u_*, and $0.7P_{u}$ is 70% the experimentally obtained maximum load of specimens in the push-out tests.

The Japanese Steel Structure Association [22] takes the secant slope at the limit bearing capacity of 1/3 as the shear rigidity, which is calculated, as follows:

(3) $$K_{1/3P_{u}}=\frac{P_{u}}{3S_{z}}$$

where $K_{1/3P_{u}}$ is the slip modulus for a single concrete wedge or bolt, $S_{z}$ is the slip at a load of 1/3*P_u_*, and $1/3P_{u}$ is 1/3 the experimentally obtained maximum load of specimens in the push-out tests.

The ductility coefficient, *D*, is defined as the ratio of the limit slip value $S_{u}$, to the corresponding 70% limit load–slip value $S_{y}$, in order to reflect the working ductility of the shear connectors, as follows:

(4) $$D=\frac{S_{u}}{S_{y}}.\;$$

where $S_{u}$ is the slip amount when loading GFRP main beam and concrete slab at the same level, $S_{y}$ is the slip at a load of $0.7P_{u}$.

In Table 3, except for specimen P3-R2-S100-NR, the ultimate displacement of the other members is greater than 14.8 mm, and the ultimate displacement of the slotted perforated plate is greater than 16.1 mm. The transverse rebar improves the restraint effect of concrete in the hole, which has significant influence on the ultimate displacement of the perforated plate. The ultimate displacement of the bolt specimen is the lowest and, the higher the bolt grade is, the smaller the ultimate displacement will be. From the perspective of the ductility coefficient, the ductility coefficient of the T-shaped perforated plate is the highest, followed by the slotted perforated plate. The failure modes indicate that both of these specimens undergo ductile failure. The brittle failure of the bolt specimen has the lowest ductility coefficient, only 1/10 that of the T-shaped perforated plate and 6/25 that of the slotted perforated plate. Two different methods are used to calculate the shear stiffness; the value of ratio $K_{1/3P_{u}}$ is higher, but the shear stiffness of the T-shaped perforated plate is the greatest, followed by that of the slotted perforated plate; the bolt has the lowest shear stiffness. The shear stiffness of the T-shaped perforated plate without transverse rebar is relatively large because of its low strength.

### 4.3. Shear Capacity of Connectors

#### 4.3.1. Bolt Connectors

At present, there is no standard and specification for the calculation of the bearing capacity of FRP section concrete composite structure bolt connections. However, the calculation method of bolt connections in steel-concrete composite structure is relatively mature. Here, as a reference, the failure modes of bolt connections are classified into two types: bolt shear failure and concrete failure. In this test, the failure modes of bolt members are bolt shear failure.

The current design codes, including those promulgated by AISC [23], ACI [24], AASHTO [25] and the literature (e.g., Nguyen [26]) have provided Equation (5) for similar failure in a steel-concrete hybrid beam to predict the ultimate load of this failure mode.

(5) $$P_{Bs}=\varphi nA_{bolt}\sigma_{bolt}$$

where $P_{Bs}$ is predicted ultimate bolt shear resistance; φ is reduction factor; *n* is bolt number in one push-out specimen; $A_{bolt}$ is cross-sectional area of a bolt; and, $\sigma_{bolt}$ is tensile strength of a shear connector.

The reduction factor φ (usually 0.6–0.8) was introduced to compute the shear strength because tensile strength could not be directly used to calculate shear failure. The average of φ should have been given as 0.6 in the present study and in Nguyen et al. [26], Correia et al. [27]. Comparison of specimens with and without epoxy is that the combined use of bolts and epoxy adhesive might result in an increase in ultimate resistance of the bolts. When only bolts are used as test pieces of the connection 0.58 is recommended, the reduction factor φ was calculated by the shear connector results of B1-R2-S150, B2-R3-S100, B3-R2-S100, and B4-R4-S90 in Table 4, supplemented with data from the literatures (Nguyen 2014 [26]; Correia 2007 [27]; Zou 2018 [17]) to predicted formula validity.

#### 4.3.2. Perforated Plate

**Figure 12:**
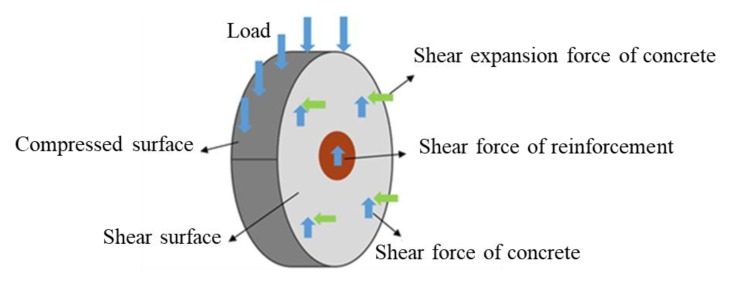
Stress of perforated plate connector.

The structure of the perforated plate connector is similar to that of the PBL connector in the steel-concrete composite structure, and its stress mechanism is similar. The transverse rebar and the concrete tenon share the bearing capacity of the joint, according to the failure mode (Figure 12).Among them, single hole of T-type perforated plate is stressed, and the double hole of slot-type perforated plate is stressed. According to the known codes, the following two codes consider both bearing capacities.

(1) Code for design of steel and concrete composite bridge (GB 50917-2013) [28]
  (6) $$N_{c}^{v}=2\alpha \left(\frac{\pi }{4}d_{1}^{2}-\frac{\pi }{4}d_{2}^{2}\right)f_{td}+2\frac{\pi }{4}d_{2}^{2}f_{vd}$$
  where $N_{c}^{v}$(*N*) is the design value of shear capacity of single hole of PBL connector (*N*); $d_{1}$ is the diameter of predrilled hole (mm); $d_{2}$ is the diameter of Transverse rebar (mm); $f_{td}$ is design value of concrete axial tensile strength (MPa); $f_{vd}$ is design value of shear strength of reinforcement, $f_{vd}=0.577f_{sd}$, $f_{sd}$ is design value of tensile strength of reinforcement (MPa); and, α is the coefficient of improvement, taking 6.1.
(2) Specifications for Design and Construction of Highway Steel-concrete Composite Bridge(JTG/T D64-01) [29]
  (7) $$V_{pu}=1.4\left(d_{p}^{2}-d_{r}^{2}\right)f_{cd}+1.2d_{r}^{2}f_{rd}$$
  where $V_{pu}$ is design value of shear capacity of single hole of PBL connector (*N*); $d_{p}$ is the diameter of predrilled hole (mm); $d_{r}$ is the diameter of transverse rebar (mm); $f_{cd}$ is axial compressive strength of concrete (MPa); and, $f_{rd}$ is design value of tensile strength of reinforcement (MPa).

In Table 5, the push out test results of the perforated plate connectors shows good consistency of JTG/T D64-01. However, the reduction coefficient should be considered for the double hole stress of the slotted perforated plate connection, which has a good enlightenment for the later research.

## 5. Conclusions

This paper presents the experimental results of the push-out tests on pultruded GFRP I-girders and concrete slabs. Three groups of push-out tests were performed to evaluate the advantages of different connectors for improving the connection between the GFRP girder and concrete slab. The failure modes of specimens with different connection types and the variation in the load–slip curves were analyzed.

The following conclusions can be drawn from this study:

(1) The failure mode of the bolt specimen is a severing of the bolt bar after the concrete around the bolt is crushed; the specimen undergoes brittle failure. There are two failure modes for the T-shaped perforated plate with transverse rebar: one is the severing of the concrete tenon and transverse rebar, the other is peeling off of the interface between the GFRP beam and the concrete; all of the specimens undergo ductile failure. The failure mode of the T-shaped perforated plate without transverse rebar is a severing of the concrete tenon; the specimen undergoes brittle failure. There are two failure modes for the slot-type perforated plate: one is a severing of the concrete tenon and transverse rebar, the other is damage to the bond layer between the opening plate and GFRP beam; and, the specimens undergo ductile failure.
(2) The load–slip curve for the bolt specimen is small at the initial stage of loading; when the ultimate load is reached, the load changes abruptly, and the sliding deformation ability becomes poor. The load–slip curve for the T-shaped perforated plate specimen exhibits an elastic stage and plastic stage, and the plastic stage retains good sliding deformation ability after reaching the ultimate load. The load–slip curve for the slotted perforated plate specimen is the same as that for the T-shaped perforated plate specimen. The difference is that there are two contact surfaces between the perforated steel bar and the opening. After one side is damaged, the other side retains some bearing capacity, which causes the load–slip curve to exhibit a platform for a period after the sudden drop.
(3) The ultimate bearing capacity of a single connection is the lowest for the bolt connection; the T-shaped perforated plate is 1.7 times greater and the slot-shaped perforated plate is 2.7 times greater. The ductility coefficient and shear rigidity of the T-shaped perforated plate are the highest, followed by the slotted perforated plate, and the bolt has the lowest values.

On the whole, the T-shaped and slotted perforated plates exhibit good anti-sliding ability and a high bearing capacity and, thus, they are good choices for interface connectors for GFRP–concrete composite beams, while bolt connectors need to be carefully chosen.

## Figures and Tables

**Table 1:** Specimen parameters.

Specimen	Connection Type	Bolt Diameter (mm)	Connection Bolts Diameter (mm)	Bolt Grade	Predrilled In The Perforated Plate	Connection Spacing	Parameter
B1-R2-S150	Bolt	10	/	8.8	/	2@150 mm	Spacing
B2-R3-S100		10		8.8		3@100 mm	Row
B3-R2-S100		10		8.8		2@100 mm	-
B4-R4-S90		6		12.9		4@90 mm	Bolt grade
P1-R2-S150	T-type perforated plate	/	6	12.9	20	2@150 mm	Spacing
P2-R3-S100						3@100 mm	Row
P3-R2-S100-NR						2@100 mm	Transverse rebar
P4-R2-S100						2@100 mm	-
G1-R3-S100	Slot-type perforated plate	/	6	12.9	20	3@100 mm	Row
G2-R2-S150						2@150 mm	Spacing
G3-R2-S100						2@100 mm	-

“B”—Bolt; “P”—T-type perforated plate; “G”—Slot-type perforated plate; “R”—Row; “S”—Spacing; “NR”—No transverse rebar. “2@150 mm”—Spacing between two rows of connections: 150 mm.

**Table 2:** Ultimate shear capacity of a single connection.

Specimen Name	Connection Type	Ultimate Shear Capacity of a Single Connection (kN)
B1-R2-S150	Bolt	37
B2-R3-S100		36
B3-R2-S100		37
B4-R4-S90		15
P1-R2-S150	T-type perforated plate	70
P2-R3-S100		58
P3-R2-S100-NR		44
P4-R2-S100		63
G1-R3-S100	Slot-type perforated plate	82
G2-R2-S150		115
G3-R2-S100		101

**Table 3:** Summary of the push-out test results.

Specimen Name	$P_{u}\;(\mathrm{kN})$	$S_{u}\;(\mathrm{mm})$	$S_{y}\;(\mathrm{mm})$	$S_{z}\;(\mathrm{mm})$	*D*	$K_{0.7P_{u}}(\mathrm{kN}/\mathrm{mm})$	$K_{1/3P_{u}}(\mathrm{kN}/\mathrm{mm})$
B1-R2-S150	297.1	6.2	1.8	0.76	3.4	1155	130..3
B2-R3-S100	435.4	5.2	1.8	0.39	2.9	169.3	372.1
B3-R2-S100	292.9	6.2	2.2	0.85	2.8	93.2	114.9
B4-R4-S90	232.1	2.7	1.7	0.95	1.6	95.6	81.4
P1-R2-S150	278.9	18.5	0.6	0.21	30.8	325.4	442.7
P2-R3-S100	346.7	22.0	0.7	0.16	31.4	346.7	722.3
P3-R2-S100-NR	174.6	0.9	0.2	0.06	4.5	611.1	825.5
P4-R2-S100	253.3	14.8	0.5	0.14	29.6	354.9	603.1
G1-R3-S100	491.5	18.6	0.8	0.30	23.3	430.1	546.1
G2-R2-S150	459.3	16.7	1.1	0.34	15.2	292.3	450.30
G3-R2-S100	409.7	16.1	0.9	0.33	17.9	315.3	413.8

**Table 4:** Validation of predicted formula validity.

Specimen Source	Specimen Name	n	Bolt Grade	$\sigma_{Bolt}$ (MPa)	$d_{bolt}$ (mm)	$P_{u}$ (kN)	φ
Present study	B1-R2-S150	8	8.8	800	10	297	0.59
	B2-R3-S100	12	8.8	800	10	435	0.58
	B3-R2-S100	8	8.8	800	10	292	0.58
	B4-R4-S90	16	12.9	1200	10	232	0.43
Nguyen [26]	H-16C1-50-35	4	/	520	16	389	0.93
	H-16C1-E-50-35	4	/	520	16	459	1.10
	H-16C2-E-50-35	4	/	520	16	454	1.08
	H-16C1-E-35-30	4	/	520	16	443	1.06
	H-16C1-E-35-20	4	/	520	16	385	0.92
	G-16C2-E-35-30	4	/	520	16	412	0.98
	H-10C1-100-50	4	/	520	10	125	0.76
Correia [27]	SCS1	4	/	800	8	68	0.42
	SCS2	4	/	800	10	157	0.63
Zou [17]	OSB-NC1	8	M4.8	400	12	225	0.62
	OSB-NC2	12	M4.8	400	12	347	0.64
	HSB-NC3	8	M6.8	600	12	325	0.60
	HSB-UHPC3	4	M6.8	400	12	355	0.65

**Table 5:** Test values and calculation values of perforated connectors in push out test.

Standard	Specimen Name	$P_{u}\;(\mathrm{kN})$	$q_{u,test\;}(\mathrm{kN})$	$q_{u,theory}\;(\mathrm{kN})$	$\frac{q_{u,test}}{q_{u,theory}}$
GB 50917-2013 [28]	P1-R2-S150	274	68.5	39.1	1.75
	P2-R3-S100	347	57.8	39.1	1.48
	P4-R2-S100	250	62.5	39.1	1.60
	G1-R3-S100	491.5	82	78.2	1.05
	G2-R2-S150	459.3	114.8	78.2	1.47
	G3-R2-S100	409.7	102.4	78.2	1.31
JTG/TD64-01 [29]	P1-R2-S150	274	68.5	56.7	1.21
	P2-R3-S100	347	57.8	56.7	1.02
	P4-R2-S100	250	62.5	56.7	1.10
	G1-R3-S100	491.5	82	113.4	0.72
	G2-R2-S150	459.3	114.8	113.4	1.01
	G3-R2-S100	409.7	102.4	113.4	0.90
